# Predictive Value of Gut Microbiome for Cognitive Impairment in Patients with Hypertension

**DOI:** 10.1155/2021/1683981

**Published:** 2021-10-06

**Authors:** Shourong Lu, Lin Shao, Yunyun Zhang, Ying Yang, Zhuo Wang, Bingshan Zhang, Jie Yu, Qiao Xu, Shuqiang Wang, Xiaorong Chen, Zhiming Yu, Yilin Ren, Kan Hong

**Affiliations:** ^1^Department of Geriatric, The Affiliated Wuxi People's Hospital of Nanjing Medical University, Wuxi 214000, China; ^2^Department of Radiology, The Affiliated Hospital of Jiangnan University, Wuxi 214000, China; ^3^Department of General Practice, The Affiliated Wuxi People's Hospital of Nanjing Medical University, Wuxi 214000, China; ^4^Department of Medicine, Wuxi Xin'an Community Health Service Center, Wuxi 214135, China; ^5^Department of Cardiovascular, The Affiliated Wuxi People's Hospital of Nanjing Medical University, Wuxi 214000, China; ^6^School of Pharmaceutical Sciences, Jiangnan University, Wuxi 214122, China

## Abstract

A connection exists between hypertension (HTN) and cognitive impairment (CI) or gut microbiota (GM) and neuropsychiatric disease. However, the link between GM and HTNCI has not been illustrated. This study endeavoured to profile the landscape of GM in HTNCI patients and evaluate the value of GM as HTNCI biomarkers. We recruited 128 patients with hypertension and assigned them to two groups of different MoCA scores. Clinical and biological data were recorded. GM composition was illustrated with 16S ribosomal RNA sequencing, and the dominant species were identified by linear discriminant analysis Effect Size (LEfSe). It showed higher abundance of *TM7* and lower abundances of *Veillonella* and *Peptoniphilus* in the HTNCI group than in the HTN without cognitive impairment (HTNnCI) group. We next clarified the link between GM and MoCA scores or HTNCI factors. KEGG analysis revealed the involvement of decreased bile secretion. An evident correlation showed up between HTNCI and *Veillonella* abundance (*P* = 0.0340). We concluded that some representative GM species, especially *Veillonella*, could predict cognitive impairment in hypertension patients, making them potential benchmarks of HTNCI.

## 1. Introduction

Dementia, often aging-related, features progressive and irreversible cognitive decline severe enough to impair the quality of life [1]. About 50 million people are suffering worldwide, with an expected annual increase of 9.9 million cases [2]. Hypertension (HTN), a culprit of cognitive impairment, is confirmed in many studies [3–7]. According to the plan of the World Health Organization, a 25% reduction in HTN prevalence by 2025 is a key effort to control cognitive decline [2]. Hypertension boosts arteriosclerotic progression in the brain, facilitating the atheroma formation and arteriolar tortuosity [8, 9]. Hypoperfusion can bring with infarction and diffuse ischemia in the periventricular and deep white matter, which favours the development of Alzheimer's disease (AD) [9]. As two leading causes of cognitive impairment, AD is manifested by early loss of episodic memory and vascular cognitive impairment (VCI) by impairment inattention, information processing, and executive function [10].

The human gastrointestinal tract populates a colony of symbiotic bacteria with abundant species and a dynamic balance. Gut microbiota (GM) is tightly involved in the physiology and pathology of humans [11]. In recent years, the imbalance of GM and its products is importantly related to the occurrence of diseases such as obesity, diabetes, hyperlipidemia, and hypertension [12–14]. In patients with blood pressure and cognitive impairment, the GM affects the human blood pressure level, brain function, and host behavior, which is related to hypertension with cognitive impairment. Studies have shown that regulating the GM can improve hypertension [15], becoming a potential key target for improving hypertension with cognitive impairment.

GM dysbiosis has a link with neuropsychiatric disorders in human and animal studies. Studies have shown characteristics of fecal microbial diversity and composition in AD patients: more bacteria eliciting proinflammatory response and less bacteria synthesizing short-chain fatty acids (SCFAs) [16, 17]. In an AD mouse model, *Verrucomicrobia* and *Proteobacteria* increase, and *Ruminococcus* and *Butyricicoccus* decrease [18]. Moreover, the tie between GM and cognitive impairment has been clarified in other diseases [19–22]. However, the profile of GM in HTNCI patients has not been elucidated. The association between GM and brain function has been explored in models using germ-free mice or animals treated with probiotics. In these studies, germ-free mice demonstrated aberrant social behaviors [23, 24] and alterations in the amygdala and prefrontal cortex [25, 26]. Other experiments based on germ-free animals found that GM was closely implicated in neurogenesis, a process critical to learning and memory [27]. Probiotics alleviated anxiety and depression in rats and mice [28]. Oral SCFAs also inhibited the decline in the function of microglia in germ-free animals [29]. Fecal microbiota transplantation could produce behavioral phenotypes [30]. However, it is a question whether these outcomes in animal experiments can be achieved in humans.

Here, we characterized the GM profile and its link with MoCA scores and HTNCI risk factors. Our findings may pluck potential diagnostic biomarkers out of GM for HTNCI.

## 2. Materials and Methods

### 2.1. Study Patients

Patients, aged >60 years, confirmed with hypertension, and treated in the Xin'an community in Wuxi city from May to October 2018 were enrolled. The exclusion criteria included the following: severe vision, hearing and speaking impairments, use of antibiotics or probiotics within the previous 6 months, diet restriction, gastrointestinal surgery, infection, mental disorders (such as schizophrenia), and severe life-threatening illnesses. The study protocol was approved by the Ethics Committee of the Wuxi People's Hospital. Each patient provided written informed consent.

### 2.2. Neuropsychological Assessment

The neuropsychological function was scored by the Montreal Cognitive Assessment (MoCA) (https://www.mocatest.org/) at 3 months after hypertension onset. A score ≥ 26 was defined as normal. To control the bias in MoCA assessment due to the education background of the patient, one point was given to the patient having education of <12 years, but not if the total score of this patient exceeded 30 points.

### 2.3. Clinical Data Collection

Basic information was collected during the clinical interview, including sex, age, education level, physique, sleep, smoking and alcohol intake, dietary habit, body mass index (BMI), and profiles of vitamin B12 and thyroid-stimulating hormone (TSH). We also prepared 128 fecal samples and stored them at -80°C.

### 2.4. Bioinformatics and Data Analysis

DNA extraction from fecal samples was performed with the FastDNA Spin Kit (MP Biomedicals, Santa Ana, CA, USA), followed by amplification of V3–V4 16S ribosomal RNA with the primers 5′-CTCCTACGGGAGGCAGCA-3′ and 5′-GGACTACHVGGGTWTCTAAT-3′. Sequencing was conducted on an Illumina MiSeq PE300 platform (Illumina, Santiago, CA, USA). After analysis on the QIIME pipeline, only high-quality sequences were retained (score > 30 and length ≥ 200 bp). Those with >97% similarity were concentrated into operational taxonomic units (OTUs) by QIIME 1.9.1 (https://qiime.org/), followed by the generation of taxonomic profiles of each OTU at five levels (phylum, class, order, family, and genus). Principal component analysis was carried out on SIMCA 14.0 (Umetrics AB, Umeå, Sweden). Candidate biomarkers were filtered out through linear discriminant analysis Effect Size (LEfSe). With an alpha value of 0.05 for both the factorial Kruskal-Wallis test among classes and the pairwise Wilcoxon-Mann-Whitney test between subclasses, the threshold on the logarithmic LDA score for discriminative features was set at 2.0. Phylogenetic investigation of communities by reconstruction of unobserved states (PICRUSt) was employed to predict the gene function of gut microbiota between groups with and without cognitive impairment. Related pathways were determined using Kyoto Encyclopedia of Genes and Genomes (KEGG) Orthology. The processing of 16S rRNA sequencing data was implemented in LEfSe and PICRUSt online (https://huttenhower.sph.harvard.edu/galaxy).

### 2.5. Statistical Analysis

Data were analyzed with GraphPad Prism V.7.0.1 (La Jolla, CA, United States), R software (V.3.4), and Adobe Illustrator CC 2015 (Adobe Systems Incorporated, California, America). Categorical variables were subjected to a chi-squared test and continuous variables to Student's *t*-test or Mann-Whitney test. The Mann-Whitney test was carried out to compare the data of the HTNCI and HTNnCI groups. Multivariate logistic regression was to determine the factors, especially representative microbes, for predicting HTNCI. The probability cut-off value was 0.05 to put in and 0.1 to put out a variable. Spearman's rank correlation was used to demonstrate the link of GM with MoCA scores and HTNCI risk factors.

## 3. Results

### 3.1. Baseline Patient Information

**Figure 1:**
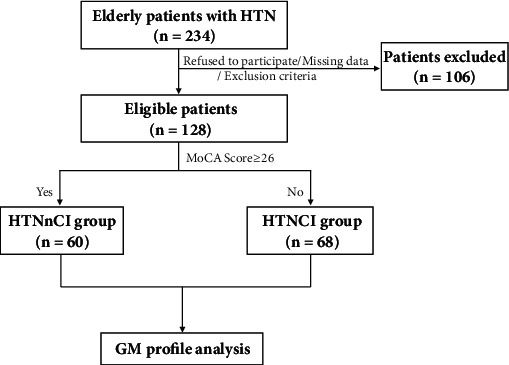
Flow chart of patient recruitment.

Recruited were 234 patients with hypertension. Excluded were 36 for unwillingness to join the study, 42 for missing data, and 28 for exclusion criteria. The 128 eligible were assigned to the HTNnCI group (*n* = 60) and the HTNCI group (*n* = 68) (see Figure 1). As shown in Table 1, significant differences were observed in sex, education level, MoCA score, stroke, and thyroid-stimulating hormone between two groups (*P* = 0.0269, 0.0161, <0.0001, 0.0191, and 0.0253, respectively), but not in age, body mass index (BMI), smoking, alcohol use, diabetes mellitus, coronary heart disease, and vitamin B12.

### 3.2. GM Profile in HTNCI Patients

**Figure 2:**
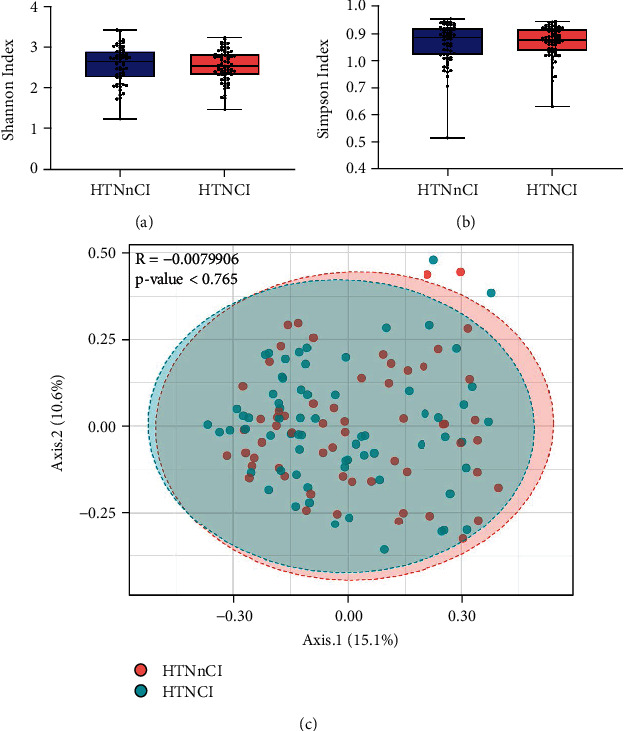
Comparison of GM components between groups' hypertension without cognitive impairment (HTNnCI) and hypertension with cognitive impairment (HTNCI). The Shannon (a) and Simpson (b) indexes were used to assess the alpha-diversity between the two groups. *P* values were determined using Mann-Whitney *U*-test. (c) Scatterplot from principal coordinates analysis showed the similar distribution between the two groups.

A total of 7825 OTUs were generated by 16S ribosomal RNA sequencing, including 14 phyla, 22 classes, 29 orders, 64 families, and 151 genera. Fecal microbiota alpha-diversity columns and the PCoA scatterplots showed no compositional difference between groups, but significant differences in the diversity of some taxa (see Figure 2).

**Figure 3:**
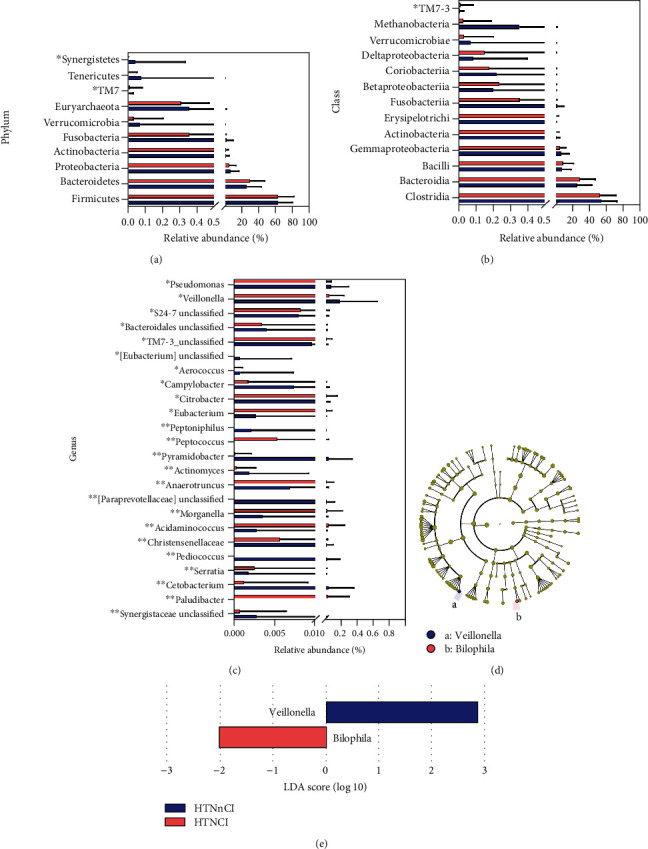
Comparison of abundances of representative GM species between two groups. The Mann-Whitney *U*-test indicated the significant between-group differences on phylum (a), class (b), and genus (c) levels. The Mann-Whitney *U*-test indicated significant between-group differences. ^∗^*P* < 0.05; ^∗∗^*P* < 0.01. (d) A cladogram of different taxonomic components between two groups. (e) Linear discriminant analysis scores showed significant between-group differences.

At the phylum level, the HTNCI group showed higher abundance of TM7 (0.00992647 vs. 0.0142756%, *P* = 0.014, see Figure 3(a)) and lower abundance of *Synergistetes* (0.0456658 vs. 0.00105054%, *P* = 0.049, see Figure 3(a)). As to classes, the HTNCI group showed higher abundance of TM7-3 (0.009926 vs. 0.014275632%, *P* = 0.014, see Figure 3(b)). At the genus level, the HTNCI group showed higher abundances of 6 genera, including *Paludibacter* (0.000 vs. 0.033162%, *P* < 0.0001, see Figure 3(c)), *Acidaminococcus* (0.002859 vs. 0.042649%, *P* < 0.0001, see Figure 3(c)), *Morganella* (0.003608 vs. 0.026179%, *P* < 0.0001, see Figure 3(c)), *Eubacterium* (0.002758 vs. 0.013626%, *P* = 0.0032, see Figure 3(e)), *S24-7 unclassified* (0.008 vs. 0.00819%, *P* = 0.0370, see Figure 3(e)), and *Peptococcus* (0.024134 vs. 0%, *P* = 0.0010, see Figure 3(c)), and lower abundances of 17 genera, including *Veillonella* (0.186036 vs. 0.05164%, *P* = 0.0340, see Figure 3(c)), *Christensenellaceae unclassified* (0.019928 vs. 0.005697%, *P* < 0.0001, see Figure 3(c)), *Anaerotruncus* (0.006951 vs. 0.020673%, *P* = 0.0001, see Figure 3(c)), Citrobacter (0.011582 vs. 0.017372%, *P* = 0.0044, see Figure 3(c)), and *TM7-3_unclassified* (0.009562 vs. 0.014074%, *P* = 0.0200, see Figure 3(c)). In LEfSe analysis, the HTNCI group showed a higher abundance of *Veillonella* and a lower abundance of *Bilophila* (see Figures 3(d) and 3(e)).

### 3.3. Predicted Functions of GM

**Figure 4:**
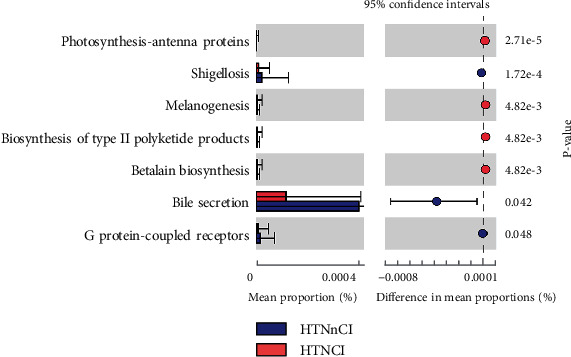
KEGG and GO analysis of gut microbiota in the HTNCI and HTNNCI groups.

We evaluated the functional differences of GM between the HTNnCI and HTNCI groups. As shown in Figure 4, in the HTNCI group, the GM was enriched in bile secretion (organismal systems, digestive system), shigellosis (human diseases, infectious diseases), and G protein-coupled receptors (environmental information processing, signalling molecules, and interaction). In HTNnCI patients, the enrichments included photosynthesis-antenna proteins (metabolism, energy metabolism), betalain biosynthesis (metabolism, biosynthesis of other secondary metabolites), biosynthesis of type II polyketide products (metabolism, metabolism of terpenoids and polyketides), and melanogenesis (organismal systems, endocrine system).

### 3.4. Correlation between GM and MoCA Score and the Risk Factors for Cognitive Impairment

**Figure 5:**
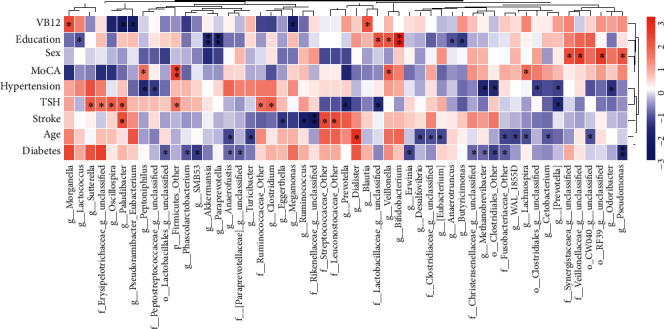
Heatmap of Spearman's rank correlation of gut microbiota with MoCA scores and risk factors for cognitive impairment. Red means positive correlation, and blue means negative correlation. ^∗^*P* < 0.05; ^∗∗^*P* < 0.01.

According to the results of Spearman's rank analysis (see Figure 5), *Lachnospira* (*P* < 0.05), *Veillonella* (*P* < 0.05), *Firmicutes_Other* (*P* < 0.01), and *Peptoniphilus* (*P* < 0.05) levels were positively associated with the MoCA score. Moreover, *Paludibacter* demonstrated a positive correlation with TSH (*P* < 0.05) and stroke (*P* < 0.05), but a negative correlation with vitamin B (*P* < 0.05). *Prevotella* was negatively correlated with TSH (*P* < 0.05) and hypertension (*P* < 0.05). In addition, *Methanobrevibacter* was negatively associated with diabetes (*P* < 0.05) and hypertension (*P* < 0.05). *Prevotella* was negatively correlated with TSH (*P* < 0.05), while *Clostridium* (*P* < 0.01), *Paludibacter* (*P* < 0.05), *Oscillospira* (*P* < 0.05), and *Sutterella* (*P* < 0.05) were all positively associated with TSH. Taken together, GM might harbor biomarkers sensitive to HTNCI.

## 4. Discussion

We drew a profile of GM in HTNCI patients in the present study. GM exhibited no significant difference in GM diversity in HTNCI patients, but in abundances of various GM components. The low abundance of *Veillonella* was detected in the HTNCI group, and its potential to predict cognitive impairment was verified in LEfSe analysis. Moreover, we associated GM profile with MoCA score and HTNCI risk factors, such as education, sex, stroke, hypertension, diabetes, vitamin B12, and TSH. Interestingly, *Veillonella* also showed an ability to discriminate HTNCI. It is suggested that GM might contain efficient biomarkers for HTNCI.

In this study, sex, education, stroke, and TSH showed between-group differences and close associations with GM. Positive correlations between hypertension and cognitive impairment and GM dysbiosis have been determined [31, 32]. Besides, high TSH may increase blood pressure to facilitate cognitive impairment [33]. This mechanism may involve the upregulation of proinflammatory cytokines, endothelial damage, and subsequent neurotoxic effects [34]. Thyroid diseases, either subclinical or clinical, interact with cardiovascular disease. A connection has showed up between thyroid function and AD [35–38]. As a typical feature of AD, *β*-amyloid mediates neurotoxicity through various mechanisms, such as inhibiting acetylcholine activity in the cortex and hippocampus [39, 40]. It also shows that thyroid function changes with systemic oxidative stress [41]. Our results supported some epidemiological studies in which multiple factors associated with HTNCI had been identified, including TSH, stroke, and education.

A previous study has hinted that GM may be associated with hypertension [32, 42–44]. In this study, higher abundances of *TM7* and *TM7-3* were found in HTNCI patients than in HTNnCI patients. Another study has indicated that the enrichment of *TM7* occurs with gut dysbiosis and contributes to inflammation [45]. Moreover, *TM7* is associated with a compromised intestinal barrier and elevated intestinal immune infiltration [46]. Evidence supports that TM7 phyla and *Proteobacteria* participate in the pathogenesis of cognitive impairment [47, 48]. Genera *Paludibacter* are positively associated with the course of Parkinson's disease [49], which is consistent with our study. Thus, all the evidences suggest the interplay between GM and HTNCI.

Besides, we presented that HTNCI involved several KEGG pathways. Bile secretion is progressively reduced in HTNCI patients. Cholesterol metabolism in the liver is closely linked with AD [50]. In fact, many genes responsible for cholesterol metabolism (e.g., BIN1 and CLU) contain susceptibility loci of AD [51, 52]. Cholesterol is decomposed by bile acids (BAs). Mounting evidence has associated immune dysregulation with AD pathology. Some immune-related genes have been discovered as risk genetic variants in AD [51, 53]. These immune-related genes may drive AD through regulating BA metabolism or GM. For example, ABI3 and MEF2C act in immune reaction upon proinflammatory stimuli from microbes [54, 55]. There is growing evidence supporting the tight connection of intestinal microbiota with the performance of the central nervous system. The central and enteric nervous systems communicate through the “gut-brain metabolic axis,” but the mechanisms hide to be clarified [56–58]. This axis is indispensable for metabolic pathways through which GM regulates metabolic activities [59]. Intestinal bacterial composition is associated with a gallery of neurological disorders [60–62]. Liver disease may aggravate cognitive dysfunctions, even leading to AD [63].

A link was set up between GM and MoCA scores or HTNCI factors. Spearman's analysis showed that *Veillonella* abundance rose as the MoCA score dropped. Previous studies have revealed that the abundance of *Veillonella* decreases in the case of cognitive impairment [64]. *Veillonella* species, as a harmless or even beneficial gram-negative anaerobic coccus, colonizes the mouth after one's birth [65]. *Veillonella* is also the main species in the gut to produce propionic acid in human GM [66]. In animal models of AD, SCFAs have shown abilities to enhance learning and memory function [67], exert neuroprotection and guard neuroplasticity, shrink *β*-amyloid plaques, and inactivate microglia [68]. Chen et al. have uncovered that fecal microbiota transplantation (FMT) could cure infarct and cerebral edema and rescue cognitive function in rats with ischemic stroke [69]. FMT can also raise the level of SCFAs, making it a potential treatment for AD [70]. However, more studies should be conducted to answer whether HTNCI arises from the scarcity of SCFAs-producing bacteria. *Bilophila* belongs to *Desulfovibrionaceae*, a type of proinflammatory bacteria that induce LPS accumulation during inflammatory response [71]. High-level *Desulfovibrionaceae* is also implicated in psychiatric disorders [72, 73], hinting at the association of *Bilophila* with HTNCI. This study provided that some species in GM could predict HTNCI, with an accuracy expected higher if combined with other valuable biomarkers.

Several limitations also exist in this study. Our results might be biased by some unstandardized variables, and the significance of the MoCA score might be overemphasized. Clinical scales should be employed to testify our results. In addition, we did not evaluate the GM profiles over a long period. Besides, the sample size was still not enough. Future experiments with larger gender-matched samples are needed.

The study has also its strengths. First, this is the first that reports the profile of the GM in HTNCI patients. Second, we constructed broader connections between GM and HTNCI risk factors. Third, this study laid a roadmap to explore reliable predictive biomarkers for HTNCI.

## 5. Conclusion

In summary, the abnormal structure of GM is associated with HTNCI. Some species in GM, especially *Veillonella*, might be adopted to predict HTNCI.

## Figures and Tables

**Table 1:** Baseline information of the HTNCI and HTNnCI groups.

Parameters	HTNnCI group (*n* = 60)	HTNCI group (*n* = 68)	*P* value
Age (years)	68.23	69.56	0.1233
Gender (male/total%)	51.67	32.35	0.0269
Low education	2.43	2.15	0.0161
BMI (kg/m^2^)	24.75	25.40	0.2179
Smoking (still smoking/total%)	30.00	25.00	0.6908
Alcohol (still drinking/total%)	25.00	16.18	0.3591
MoCA score	27.27	19.16	<0.0001
Diabetes mellitus	0.30	0.29	0.9426
Coronary heart disease	0.083	0.18	0.1232
Stroke	0.10	0.26	0.0191
Vitamin B12 (pg/mL)	263.00	230.50	0.3288
TSH (mU/L)	4.91	10.02	0.0253

TSH: thyroid-stimulating hormone.

## Data Availability

The analyzed data sets generated during the study are available from the corresponding authors on reasonable request.
